# Anorexia nervosa and autoimmune comorbidities: A bidirectional route?

**DOI:** 10.1111/cns.13953

**Published:** 2022-09-16

**Authors:** Maria M. Sirufo, Lina M. Magnanimi, Lia Ginaldi, Massimo De Martinis

**Affiliations:** ^1^ Department of Life, Health and Environmental Sciences University of L'Aquila L'Aquila Italy; ^2^ Allergy and Clinical Immunology Unit Center for the Diagnosis and Treatment of Osteoporosis Teramo Italy

**Keywords:** anorexia, autoimmunity, immunity, microbiome, neuropsychoimmunology

## Abstract

Immunological dysfunctions in eating disorders have recently gained increasing scientific attention. Furthermore, the reciprocal association between anorexia and autoimmune diseases is of particular interest and suggests a role of autoimmunity in the pathogenesis of eating disorders. Anorexia nervosa (AN) and autoimmune diseases are linked by a bidirectional relationship based on common immunopathological mechanisms. In this review, in addition to reporting the numerous cases described in which autoimmune disorders are associated with anorexia or vice versa, we summarize the many aspects of this relationship between the immune system (IS) and AN. We describe how the microbiota affects the IS, disrupts gut‐brain communication, and possibly triggers eating disorders. We also describe the shared immunological pathways of autoimmune and eating disorders and in particular the occurrence of disrupted T cell tolerance and autoantibodies in AN. The described observations represent the starting point for possible, future research directions.

## Graphical abstract

Anorexia nervosa and autoimmune diseases are linked by a bidirectional relationship based on common immunopathological mechanisms. A possible link is the intricate mechanisms that intertwine the gut microbiome, nervous system and immune system: microbiota affects the immune system and disrupts the gut‐brain communication.

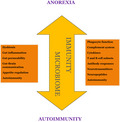

## 1 INTRODUCTION

Anorexia nervosa (AN) is a worrying and severe psychiatric disease diagnosed when these three conditions are met: significantly low body weight in relation to age and sex, developmental trajectory and physical health of the patient, and an intense fear of weight gain and a disturbed body perception.^1^ Medical complications affecting all body systems are frequently associated with increased mortality.^2^ In adolescence, AN is the third most frequent chronic disturbance and has the highest mortality among all psychiatric conditions. The disease occurs worldwide among males and females of all ages and is associated with a mortality risk increased by five times or more. Its overall incidence over the past decades is considered stable. In persons aged <15 years incidence has increased, but it is unclear whether this increase depends on earlier age of onset or earlier detection. AN has a lifetime prevalence rate of up to 0.3% among males and 4% among females^3^ and is also associated with a high incidence of coexisting psychiatric conditions, treatment resistance, and suicidal risk.^4^ We still know little about the exact etiology of AN and this limits the possibility of developing new therapeutic approaches. Understanding the biological correlates of the disease that contribute to the development and maintenance of the disorder is crucial for possible targeted interventions.^5^, ^6^, ^7^, ^8^, ^9^ A new unifying vision of eating disorders comes to us from psycho‐neuro‐immuno‐endocrinology, which integrates knowledge derived from the psychological and biomedical sciences.^10^

Psychological theories of anorexia reigned for generations,^11^ but now researchers are working to untangle the biology of the disease and a succession of discoveries suggests that the biological roots of the disease run deep. Curiously, some psychiatric disorders have been linked to excessive activation of the immune system (IS). The release of soluble inflammatory mediators and the mobilization of immune cells can also be stimulated by adequate psychological stressors.^12^ Both functional and structural brain alterations^13^ such as brain volume loss, astrocyte reduction, and inflammation^14^ are commonly related to AN. We have a lot of evidence of how the IS, along with neurochemical and hormonal responses, can significantly influence central nervous system (CNS) stress circuitry, especially through inflammation molecules. It is intuitive that the brain and the IS are the ones mainly responsible for regulating relations with the external environment and have evolved together with the aim of preserving the internal homeostasis of the organism. Therefore, these are systems strictly interconnected in a bidirectional way allowing a balanced and safe relationship of the organism with the environment.^12^ AN is associated with a mild inflammatory state, alterations in the immune response, the production of autoantibodies, and occurs during autoimmune diseases, but this is also true in the opposite sense: those affected by autoimmune disorders present similar pathophysiological conditions and can develop anorexia. The aim of our work is to bring together the current knowledge on the neuroimmune interaction in AN with new interesting therapeutic perspectives as opened up by the hypothesis of autoimmune mechanisms.

## 2 THE BIDIRECTIONAL RELATIONSHIP BETWEEN THE IS AND AN

**FIGURE 1:**
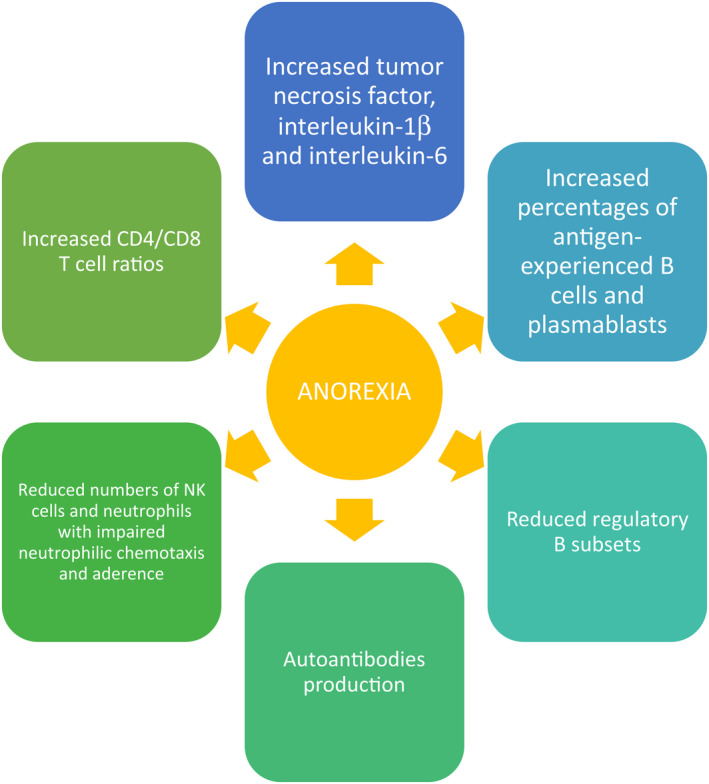
Immune changes associated with anorexia nervosa

The existence of a relationship between IS and eating disorders is indubitable and many studies have dealt with it,^10^, ^15^, ^16^, ^17^, ^18^, ^19^, ^20^, ^21^, ^22^, ^23^, ^24^, ^25^, ^26^, ^27^, ^28^, ^29^, ^30^ however, these have several limitations, their results are often conflicting and many aspects are still to be investigated. The IS is dysregulated in anorexic patients as strongly suggested by the proinflammatory cytokines profile and other markers of central and peripheral inflammation. Inflammatory and autoimmune diseases are interestingly associated with the risk of developing bulimic syndromes. The relationship between IS and nutrition can be studied from two opposite points of view. This relationship was initially investigated by considering the effect on the IS of an inadequate nutritional intake, but subsequently, it became evident that conditions of primary or secondary dysregulation of the immune state (such as in the course of infections or autoimmune diseases) induce anorexia. Malnutrition deprives the IS of several components essential to generating an effective immune response.^17^ Immunocompetence and nutritional status are closely linked.^18^ The most consistent alterations are observed in the phagocyte function, complement system, cytokine production, mucosal secretory antibody response, antibody affinity, and cell‐mediated immunity.^17^ Immune changes and increased risk of infection seem to be associated with chronicity of AN.^19^ Immune system changes in AN (Figure 1) were reported to be not so severe as those observed in malnutrition secondary to somatic diseases.^20^ Leukopenia, polycythemia, and thrombocytopenia are reported in adolescent and young adult males with AN.^30^ Peripheral blood (PB) of anorexic subjects shows elevated levels of proinflammatory cytokines. A mild inflammatory status characterizes AN with an increase of tumor necrosis factor (TNF), interleukin(IL)‐1α, and IL‐6^5^ together with modulation of cellular components of the adaptative and innate IS. Interleukin‐6 and other cytokines have a leading role in the anorexia of aging, linked to apoptosis.^31^, ^32^ Recently Tyszkiewicz‐Nwaforet al. showed that the fasting levels of interleukins‐1 (IL‐1), 6 (IL‐6), and 8 (IL‐8), C‐X‐C Motif Chemokine Ligand 1 (CXCL1), C‐X‐C Motif Chemokine Ligand 9 (CXCL9), metalloproteinases 8 (MMP8) and 9 (MMP9), cathepsin S (CTSS), fibroblast growth factor 2 (FGF2), and granzyme B (GrB) are diminished in the initial phase of restrictive AN in adolescents in the first year of the disease.^20^ These data suggest the absence of a pro‐inflammatory state in these subjects. Furthermore, they show that some immune‐related protein alterations may be associated with changed neuropeptides, primarily leptin, its receptors, and resistin. NK cells and neutrophils are reduced in numbers while neutrophilic chemotaxis and adherence are impaired. PB CD4/CD8 T cell ratios are increased as CD8^+^ T cells show to be diminished among overall T cells.^21^, ^22^ No changes in the proportion of regulatory T cells (T_regs_) are described in AN. Studies on T cell proliferation do not show univocal data as it can be equivalent, increased, or reduced, depending on the mitogens used. Regarding the number and percentage of B cells in anorexic subjects, there do not seem to be significant variations compared to healthy controls. Only Elegido et al.^21^ reported an increase of B cells in anorexic adolescents, but with comparable percentages. We can learn more about the cellular B compartment by studying the composition of the B cell compartment on the basis of maturation immunophenotype. The efficiency of the IS in subjects suffering from AN has also been studied through the evaluation of the response to vaccines,^33^ but no significant differences emerged with respect to the normal weight population both with respect to the flu vaccine^34^ and H1N1 vaccination.^35^ Subsequently, the view from the opposite perspective developed, that is how immunological alterations could generate anorexia, known to be one of the most characteristic symptoms in the course of several diseases, derived mainly from pharmacological treatment and inflammatory processes.^23^ Even if we are still very far from understanding them, there is more and more evidence suggesting a relevant role of immunological mechanisms in the genesis of AN. A potential primary immunologic defect contributing to the development of AN is conceivable.^36^ Control of the immune function of the neuroendocrine system is recognized and several studies support the relevance of immunoendocrine factors in the aetiopathogenesis of AN.^24^ The crucial role of the interaction between neurotransmitters, neuropeptides, and cytokines in the pathogenesis of AN has been suggested since 1996.^25^ Increased risk of developing eating disorders in association with infections has been shown. Anorexia can initially be part of the body's defensive armamentarium and then becomes an independent disease. The remodeling of the intestinal microbiota is among the pathophysiological hypotheses.^26^ It seems that the dysbiosis associated with infections is similar to that observed in the course of AN, however, it is yet to be defined whether the infection represents a significant trigger along with others in subjects predisposed for genetic and/or epigenetic reasons.^27^ Changes in neurotransmitters, neuropeptides, and cytokines and their interactions have a leading role in the pathophysiology of anorexia. The fundamental role of cytokines in the modulation of inflammation and controlling infections is acknowledged, as well as in the regulation of neurotransmitter systems, neuroplasticity, and neuroendocrine functioning.^20^, ^28^ In conclusion, a dysregulated IS seems characteristic of AN while it is becoming increasingly intriguing to consider that the IS may actually be causal in the pathogenesis and maintenance of AN.

## 3 THE MICROBIOTA AFFECTS THE IS, DISRUPTS THE GUT‐BRAIN COMMUNICATION, AND POSSIBLY TRIGGERS EATING DISORDERS

The gut microbiome has a leading role in regulating metabolism, gastrointestinal symptomatology, appetite, behavior, and mood, and one of healthy individuals, may diverge from that of AN patients for a number of potential avenues.^37^ The intricate mechanisms that intertwine the gut microbiome, nervous system, and IS are implicated in the pathophysiology of eating disorders.^29^ Recent studies suggested that the brain‐gut‐microbiota axis is a complex system that plays a crucial role in the occurrence and development of central nervous system diseases.^38^ Through several interactions, the microbiota may impact both autoimmune diseases and AN. These include innate and adaptive immunity, epigenetic alteration of protein expression, proteostasis, hematopoiesis, coagulation, hormone secretion, autonomic nerve system, and metabolism.^39^ Several observations demonstrated that via the gut‐brain axis the microbiota acts on the neurobiological, inflammatory, and immune pathways implicated in AN and comorbidities. The synthesis of neurotransmitters by the intestinal microflora is essential in the gut‐brain interplay. The study of microbiota is a research field of increasing interest and it was shown that a contribution to the development of AN could come from the association of a specific genetic susceptibility with dysbiosis. Perturbations of the intestinal microbiota that could represent disease biomarkers have been identified in multiple psychiatric disorders.^40^ Furthermore, recent stroke research suggested that a specific set of inflammatory microbiota exists in central and peripheral organs and can serve as a disease biomarker and a therapeutic target.^41^ In the pathophysiology of AN, dysbiosis disrupts proper communication between the gut and the brain, promoting gut inflammation, altering intestinal permeability, and triggering immune reactions in the regulation center of hunger/satiety. Microbiota changes alter the hunger/satiety regulatory system, mood, and the hypothalamic–pituitary–adrenalaxis. The gut microbiota changes significantly in quantity, quality, and composition in subjects with AN following the changes in weight. Assimilation and accumulation of calories from food and changes in the IS are a consequence of the reduced intestinal microbial biodiversity.^42^ Lower alpha diversity is observed during weight loss with the increase of Enterobacteriaceae, Actinobacteria, *Methanobrevibactersmithii*, and Bacteroides and a reduction of short‐chain fatty acids (SCFAs) and Firmicutes. Weight recovery corresponds an increase of SCFAs and recovery of the Firmicutes/*Bacteroides* ratio.^43^ Interestingly, *Escherichia coli* produces large quantities of caseinolytic peptidase B (ClpB) which has a role in the stimulation and autoimmune response,^44^, ^45^ capable of modifying feeding behavior by acting on the melanocortin system. In AN, altered microbiota produces neuropeptide‐like proteins able to stimulate endogenous satiety and appetite hormones and induce an immunoglobulin cross‐reaction. This is the case of autoantibodies against the α‐melanocyte‐stimulating hormone (α‐MSH) acting directly on the arcuate nucleus and on the regulation of appetite. The increase in anti‐α‐MSH antibodies observed in subjects with AN correlates with psychopathological symptoms and high stress.^43^ Changes in the gut microbiome and IS can serve as biomarkers of increased risk of developing AN as well as maintaining and exacerbating incorrect eating habits.^46^ The role of the microbiota and its action on the IS in the pathogenesis of various diseases is ultimately evident. Changes in the microbiota and some specific immunological profiles can become valuable biomarkers of disease and useful therapeutic targets.

## 4 DISRUPTED T CELL TOLERANCE AND AUTOANTIBODIES IN AN

A recent study, showing the presence of immune dysregulation in psychiatric disorders, speculates on the role of autoimmunity in these conditions. In particular, it was underlined as eating disorders, similar to several other psychiatric conditions, are associated with the presence of autoantibodies.^15^, ^47^ A relatively large number of brain antigens are implicated in autoimmune diseases^48^ and this suggests evidence of a possible co‐evolution of IS and CNS. Thus, in several autoimmune diseases including rheumatoid arthritis, psoriasis, multiple sclerosis or diabetes, enzymes or receptors for the neurotransmitters GABA (GAD65) and acetylcholine (AchR) are targeted, as well as tyrosine hydroxylase, laminin, and myelin. It is intriguing that only a small subset of an enormity of possible antigens are recognized to be responsible for such autoimmune conditions and that it is easy to find them in large amounts in the CNS. It can be hypothesized that many of these super autoantigens capable of overcoming the regular inhibitory limitations of the IS are hosted in the CNS and it is suggestive to think that this is due to the evolutionary pressures that have bi‐directionally shaped the immunity‐brain cooperation. It is also conceivable that, at least in part, our communities, increasingly interconnected, and our growing social ties have driven the co‐evolution of the IS and of the brain, while the unmet social needs represent some of the new stressors of our psychosocial world. The increase in immunological defensive strategies and their complexity has also increased the possibility of weaknesses for the onset of possible complex pathologies. Acres MJ et al.^49^ have suggested autoimmunity underlying AN. They hypothesized the production of autoantibodies directed against regulatory peptides and hypothalamic neurons, cross‐reacting with microbial antigens, due to a delayed exposure to common microorganisms. These autoantibodies would induce an appetite disorder with reduced food intake. In subjects with AN, the presence of IgG, IgA, and IgM autoantibodies against several appetite‐regulating peptides is detected. The latter show antigenic homologies with the gut microbiome and a positive correlation were found between AN and the presence of autoantibodies to alpha‐melanocyte stimulating hormone (α‐MSH). IgM antibody levels against α‐MSH are increased in patients with eating disorders compared to controls.^16^ However, the study reports the lack of data on the prevalence in adults with eating disorders of cerebrospinal fluid/serum autoantibodies.^15^ So the regulation of eating behavior depends on the gut‐brain‐adipose tissue (AT) peptides and the presence of neutralizing autoantibodies may result in AN.^50^ Psychiatric disorders have been associated with the presence of neural autoantibodies, although the significance of this association often remains difficult to explain.^15^, ^51^ Autoantibodies, directed against key appetite‐regulating peptide hormones or neuropeptides, were found in healthy subjects while psychopathological traits of people affected by eating disorders correlate with the amount and affinities of autoantibodies against anorexigenic and orexigenic neuropeptides. The development of AN could be triggered by access to the brain centers of these high‐affinity autoantibodies.^52^ Furthermore, an immuno‐reactivity toward primate hypothalamic neurons in serum from AN subjects was found by Escelsior et al.^53^ Diet has an impact on autoimmunity.^54^ Increased gut permeability to food antigens and the potential reduction of oral tolerance could justify the presence of a mild inflammatory state and the increased possibility of developing autoimmunity.^19^ Even small alterations in the intrinsic signaling programs of B lymphocytes are sufficient to trigger a systemic autoimmune process through the disruption of T cell tolerance. The increase in autoreactive mature B lymphocytes is a consequence of altered signaling programs of B cells that modulate their negative or positive selection.^55^ AN patients show increased percentages of antigen‐experienced B cells and plasmablasts while regulatory B cell subgroups are reduced. Furthermore, these latter cells have a strong relationship with body composition suggesting their leading role in the immunopathogenetic mechanism of AN. These alterations in B lymphocyte subsets may at least partially explain the production of autoantibodies.^19^

## 5 THE SHARED IMMUNOLOGICAL PATHWAYS OF AUTOIMMUNE AND EATING DISORDERS

**FIGURE 2:**
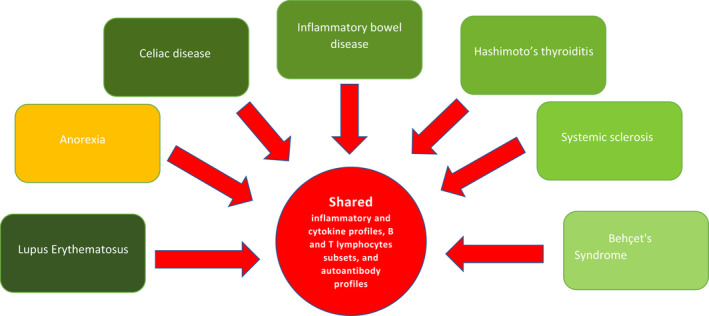
Common immunological changes of AN with autoimmune diseases. The link between eating disorders and autoimmune diseases is based on shared immunological mechanisms: inflammatory and cytokine profiles, B and T lymphocytes subsets, and autoantibody profiles. Moreover, the interplay between the gut microbiome, immune regulation, and sex hormones thus provides one potential, the complex mechanism underlying eating disorders and explains the partly shared etiopathogenesis of eating disorders and autoimmune diseases.

A high prevalence of autoimmune diseases is reported among patients with eating disorders.^53^ Raevuori et al.^56^ believe that shared immunological pathways link autoimmune and eating diseases (Figure 2) and that, at least in a subset of subjects, autoimmunity triggers and maintains the eating disorder. The association between various autoimmune diseases and anorexia is described (Table 1). Hyla‐Klekot et al.^57^ described a case of associated anorexia and juvenile systemic lupus erythematosus. Like other authors before them, they suggest a common origin of the two diseases on the basis of their coexistence and the resolution of anorexia as a consequence of the immunosuppressive treatment. Other similar cases have been described in the past and AN was considered a possible onset manifestation of lupus erythematosus.^58^, ^59^, ^60^, ^61^ Anorexia could also be one of the presenting symptoms of lupus with gastrointestinal involvement.^62^ Celiac and Inflammatory bowel diseases may trigger the development of eating disorders.^63^, ^64^, ^65^ Coexistence of Hashimoto's thyroiditis and AN are also reported.^66^, ^67^ Dell'Osso et al.^68^ recently reported the association of Behçet's Syndrome with AN.

**TABLE 1:** The references listed in the table highlight the association between various autoimmune diseases and anorexia

Hyla‐Klekot L. et al.	Anorexia nervosa and juvenile lupus erythematosus in a 16‐year‐old female patient—common disease origin or random coincidence?	Cent Eur J Immunol	2021
Toulany A. et al.	Chicken or the egg: anorexia nervosa and systemic lupus erythematosus in children and adolescents.	Pediatrics	2014
Bambery P. et al.	Anorexia nervosa in a patient with systemic lupus erythematosus.	Rheumatol Int	1987
Sloan D. et al.	Anorexia nervosa complicates systemic lupus erythematosus (SLE).	Ir Med J	1998
Dalbeth N. and Callan M.	Arthritis and anorexia?	Lancet	2002
Trapani S. et al.	Gastrointestinal and hepatic involvement in pediatric systemic lupus erythematosus.	Clin Exp Rheumatol	2021
Tokatly Latzer I. et al.	Disordered eating behaviors in adolescents with celiac disease.	Eat Weight Disord	2020
Ilzarbe L. et al.	Inflammatory bowel disease and eating dsisorders: A systematized review of comorbidity.	J Psychosom Res	2017
Blanchet C. and Luton JP.	Anorexie mentale et maladie de Crohn: intrications et difficultés diagnostiques. Trois cas [Anorexia nervosa and Crohn disease: diagnostic intricacies and difficulties. 3 cases].	Presse Med	2002
Pehlivantürk Kızılkan M. et al.	An adolescent boy with comorbid anorexia nervosa and hashimoto thyroiditis.	J Clin Res Pediatr Endocrinol	2016
Smalls‐Mantey A. et al.	Hypothyroidism due to Hashimoto's thyroiditis masked by anorexia nervosa.	Int J Eat Disord	2015
De Martinis M. et al.	Raynaud's phenomenon and nailfold capillaroscopic findings in anorexia nervosa.	Curr Med Res Opin	2018
Dell'Osso L. et al.	Subthreshold autism spectrum in a patient with anorexia nervosa and Behçet's syndrome.	Case Rep Psychiatry	2020

AN patients with Raynaud's phenomenon could show an early scleroderma pattern by studying their microcirculation by videocapillaroscopy.^69^, ^70^ Association of scleroderma and anorexia have been reported^71^ and the presence of a common clinical and capillaroscopic picture between systemic sclerosis^72^ and AN is very intriguing. These observations strengthen the hypothesis of shared pathogenetic mechanisms that could also trigger each other. These disease associations can also lead to misdiagnosis. In both conditions, AN and autoimmune diseases, inflammatory and cytokine profiles, B and T lymphocyte subsets, and common autoantibody profiles are reported. Several other shared factors, such as being a female, abnormal levels of estrogen preponderance, metabolic changes mediated by adipokines such as leptin and adiponectin, elevated cytokines, and lower abundance or diversity of intestinal microbiota, potentially influence the relationship between AN and autoimmunity.^73^, ^74^, ^75^ Furthermore, cortisol levels are dysregulated in AN and the same molecule is often included in the therapeutic regimen for autoimmune diseases. Moreover, a genetic overlap was suggested between psychiatric disorders and several autoimmune disturbances.^76^ Dysregulation of the inflammatory system characterizes autoimmune diseases and AN, with an increase of pro‐inflammatory cytokines. In the latter, an alteration of the neurotrophic system is also observed.^77^ All the cases described above, support the hypothesis of common pathophysiology between autoimmune diseases and eating disorders, which represent a mutual alert and each confers an increased risk for the other.^78^ During the COVID‐19 pandemic, there was a significant increase in the number of cases of AN^79^, ^80^, ^81^ and also in the development of autoimmune phenomena.^82^, ^83^, ^84^ Similar to other viruses, SARS‐CoV‐2 seems capable of triggering autoimmune reactions.^85^ The dysregulation of the IS induced by COVID‐19 induces the development of autoimmune phenomena even if related mechanisms are not yet known.^86^ Without diminishing the importance of psychosocial factors, it could therefore be hypothesized that this increase in the burden of EDs could also be linked to the increase in self‐reactivity of an imbalanced IS as a consequence of the infection with SARS‐CoV‐2.

## 6 CONCLUSIONS

**FIGURE 3:**
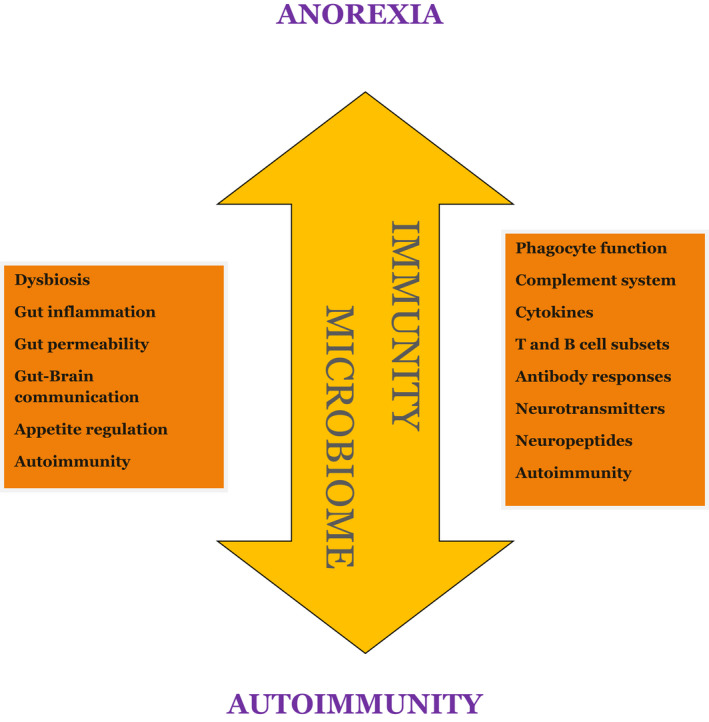
There is a bidirectional relationship between autoimmunity and anorexia nervosa: autoimmune diseases and eating disorders give each other a mutual increase in risk. Altered immunity and dysbiosis have leading roles in these processes.

AN and autoimmune diseases are linked by a bidirectional relationship based on common immunopathological mechanisms (Figure 3). There is evidence that those suffering from autoimmune diseases can develop anorexia and vice versa it would seem that those suffering from AN could develop an autoimmune disease. The presence of a mild inflammatory state, the alterations of the immune response, and the production of autoantibodies would be the common pathogenetic substrate for these conditions. It is an intriguing topic full of suggestions. We summarized and highlighted current knowledge to provide impulse and amplify the stimulus for new research. To date there is a complete lack of systematic studies able to precisely define the common immunopathogenetic mechanisms and the links between autoimmunity and eating disorders and establish the mutual risk of passing from one disease to the other. Several data further encourage a reconceptualization of AN as a psycho‐neuro‐endocrine‐immune disorder. Elucidating the immune component is a critical direction for future research, and paying attention to both neuro‐psychiatric and immune components may be key to improving outcomes.

## AUTHOR CONTRIBUTIONS

M.M.S., L.M.M, L.G., and M.D.M. contributed equally to the work. All authors read and approved the final manuscript.

## CONFLICT OF INTEREST

The authors declare that they have no competing interests.

## Data Availability

Data sharing not applicable to this article as no datasets were generated or analysed during the current study.
